# Pain cognitions and impact of low back pain after participation in a self-management program: a qualitative study

**DOI:** 10.1186/s12998-022-00416-6

**Published:** 2022-02-21

**Authors:** Lise Joern, Alice Kongsted, Line Thomassen, Jan Hartvigsen, Susanne Ravn

**Affiliations:** 1 Department of Sports Science and Clinical Biomechanics, University of Southern Denmark, Campusvej 55, 5230 Odense M, Denmark; 2 Chiropractic Knowledge Hub, 5230 Odense M, Denmark

**Keywords:** Back pain, Pain perceptions, Common sense model, Self-efficacy, Self-management

## Abstract

**Background:**

Benefits from low back pain (LBP) treatments seem to be related to patients changing their pain cognitions and developing an increased sense of control. Still, little is known about how these changes occur. The objective of this study was to gain insights into possible shifts in the understanding of LBP and the sense of being able to manage pain among patients participating in a LBP self-management intervention.

**Methods:**

Using a qualitative study and a content analytic framework, we investigated the experiences of patients with LBP who participated in ‘GLA:D^®^ Back’, a group-based structured patient education and exercise program. Data were generated through qualitative semi-structured interviews conducted between January 2019 and October 2019. Interviews focused on experiences with pain and were analysed using a thematic analytical approach. The Common Sense Model and self-efficacy theory formed the theoretical framework for the interpretations. Participants were sampled to represent people who were either dissatisfied or satisfied with their participation in GLA:D^®^ Back. Fifteen participants aged 26–62, eight women and seven men, were interviewed from February to April 2020.

**Results:**

Four main themes, corresponding to the characterisation of four patient groups, were identified: ‘Feeling miscast, ‘Maintaining reservations', ‘Struggling with habits’ and ‘Handling it’. The participants within each group differed in how they understood, managed, and communicated about their LBP. Some retained the perception of LBP as a threatening disease, some expressed a changed understanding that did not translate into new behaviors, while others had changed their understanding of pain and their reaction to pain.

**Conclusions:**

The same intervention was experienced very differently by different people dependent on how messages and communication resonated with the individual patient's experiences and prior understanding of LBP. Awareness of the ways that individuals’ understanding of LBP interact with behaviour and physical activities appear central for providing adaptive professional support and meeting the needs of individual patients.

**Supplementary Information:**

The online version contains supplementary material available at 10.1186/s12998-022-00416-6.

## Background

Low back pain (LBP) is the most prevalent musculoskeletal condition and in top ten of all conditions responsible for years lived with disability worldwide [1, 2]. There is an obvious need to reduce the burden of LBP in terms of the disability and poor quality of life experienced by people who live with severe LBP and in terms of the substantial costs to societies [3].

Pain cognitions and individuals’ perceived ability to influence their pain may explain how LBP leads to disability [4, 5]. Also, improved pain self-efficacy and illness perceptions appear to be part of the mechanisms of change that explain how interventions for musculoskeletal pain conditions work [6–8].

GLA:D^®^ Back is a 10-week group-based education and exercise program that translates guideline recommendations into a clinician-delivered program for the promotion of self-management in people with persistent or recurrent LBP [9, 10]. It was developed to address a need for interventions that provide a change in a patient’s beliefs and behaviours associated with LBP through helpful explanations of pain that can replace structural or biomedical explanations, and guidance to restore normal and varied movements, as well as confidence in movement and physical activities.

The objective of this study was to gain insights into the possible shifts in the understanding of LBP and the sense of being able to manage pain among patients participating in the GLA:D^®^ Back program.

### Theoretical framework

The Common Sense Model and the self-efficacy element of the Social Cognitive Theory present the central theoretical framework used to investigate the dynamics underlying relationships between pain experience, pain perceptions, and disability in LBP [11–13]. Both theoretical frameworks emphasise the effect of personal experience on initiating and achieving change in handling one’s illness. Constructs of the Common Sense Model are mainly found to be associated with health outcomes, whereas self-efficacy has been more consistently associated with self-management behaviors [14]. Integration of the models has been proposed to address both patients’ conceptions of illness via the Common Sense Model, and their perceived ability to adopt and maintain new behaviours, i.e. self-efficacy for managing illness [15].

The Common Sense Model describes how illness beliefs affect health behaviors, i.e., that people react to a perceived threat from their symptoms or a diagnostic label in a way that makes sense based on their understanding of these [16–18]. If you, for example, understand disc degeneration as something worsened by moving the spine, it makes sense to move less.

Self-efficacy is people's beliefs in their ability to influence events that affect their lives [13, 19]. Four main sources have been described that form the basis for developing self-efficacy: Personal experience that a task or situation is manageable; vicarious experiences from observing other people in a similar situation managing it; verbal persuasion, which depends on the credibility of the source e.g. if a clinician is considered trustworthy by a patient; and finally the interpretation of physiological and emotional reactions (e.g. increased heart rate or pain provocation) can affect the individual’s confidence in task performance.

These theoretical frameworks have formed the basis for capturing participants’ processes and their attempts to understand and manage pain after having participated in GLA:D^®^ Back. The frameworks can further assist in understanding how these processes and attempts can be optimized to facilitate constructive changes.

## Methods

The qualitative study was underpinned by the theoretical framework of content analysis [20]. Data was generated through semi-structured interviews focusing on participant’s experiences, views on their pain experiences, and management of LBP after having participated in the GLA:D^®^ Back program. The exploration of the data was geared towards a systematic organization of the patients’ descriptions and views on their LBP experiences. Interviews were conducted February to April 2020. The study was reported in accordance with the Consolidated criteria for reporting qualitative research (COREQ) checklist [20].

### Participants

Participants were a purposive sample of people with persistent LBP aged above 18 years of age, who had participated in the GLA:D^®^ Back program, and who had responded to a follow-up questionnaire. GLA:D^®^ Back was offered by physiotherapists and chiropractors in private primary care clinics nationwide in Denmark. By end of 2020, approximately 4000 patients had participated in the program. Most of participants were females reporting LBP for > 1 year, and around 45% had a higher education. On average participants had moderate pain intensities and moderate disability levels at enrolment [21]. Participation was decided in a dialogue between patient and clinician and involved out-of-pocket payment [9]. To obtain a study sample with diverse experiences as reflected in their satisfaction with the program, we did purposive sampling inviting both participants who were dissatisfied and satisfied according to their scores on a 5-point satisfaction question (From ‘Not at all’ to ‘To a very large extent’) (Table 1). In this sense, the study was designed to achieve representation of the diversity of outcomes from a self-management intervention and of lived experiences of pain management. In total 15 participants aged 26–62 were interviewed. For logistic reasons participants for this study were sampled from two out of five Danish regions (Central Denmark Region and Region of Southern Denmark).

**Table 1:** Study participants’ demographics and satisfaction with GLA:D^®^ Back. M: Male; F: Female

Participant number	Gender	Age	Satisfaction with GLA:D^®^ Back participation
01	M	58	Satisfied to a very large extend
02	F	52	Satisfied to a very large extend
03	F	45	Satisfied to a very large extend
04	F	52	Satisfied to a very large extend
05	M	62	Satisfied to a very large extend
06	F	35	Somewhat satisfied
07	F	26	Somewhat satisfied
08	M	55	Somewhat satisfied
09	M	53	Somewhat satisfied
10	F	38	Somewhat satisfied
11	F	52	Not at all satisfied
12	M	39	Satisfied to a small extent
13	M	61	Satisfied to a small extent
14	M	54	Not at all satisfied
15	F	47	Satisfied to a small extent

### Data collection and analysis

Semi-structured one-on-one interviews were conducted in participants’ home (n = 6) or via telephone (n = 9) between February and April 2020. All interviews were performed by LT, a female physiotherapist (MSc PT) employed as research assistant in the GLA:D^®^ Back program who had no pre-existing relationship with the study participants. Each interview was audio-recorded and lasted between 19 and 65 min (mean 41 min). Prior to this study LT had performed 8 qualitative interviews that addressed similar aspects to this study with participants in a GLA:D^®^ Back pilot study [22]. No pilot testing of the final interview guide used for this study was performed.

A semi‐structured interview guide with open‐ended questions was used to stimulate participant’s descriptions. The self-efficacy element of the Social Cognitive Theory and The Common Sense Model constituted an implicit framework for the interview guide. The main topics were centered on participants’ descriptions of pain management before and after attending the GLA:D^®^ Back program (Additional file 1: Interview Guide).

All interviews were transcribed verbatim by LT and three student assistants into NVivo software (QSR International, version 12). The assistants marked in the transcription any doubts about what was said or how to indicate any nonverbal expressions. Transcripts were not returned to participants for comments or corrections. We then coded the data into themes and identified common themes following the six-phase analytical strategy of thematic analysis [23]. In brief the phases are phase 1: Become familiar with the data; phase 2: Generate initial codes; phase 3: Identify themes by combining group of codes with similar meaning; phase 4: Review themes; phase 5: Define themes across data set; phase 6: Write-up. Thematic synthesis was chosen, as it is a tried and tested method in qualitative research, allowing systematic identification of common or key themes across data sets, while critically checking consistency in the ways themes are identified, how they are related to the theoretical framework, and how they contribute to answering the research questions of the project [24]. The interviews and analyses were performed in Danish and quotes translated to English by LJ (Associate Professor, Ph.D.) for publication.

To ensure reliability, the analysis was carried out by involving researcher triangulation. In practice coding and identifying defining themes were first performed by the interviewer LT immediately following the interview. Coding was then performed anew by LJ ensuring that the identified codes and themes represent LT and LJ’s common understanding of the content of the interviews. The theory of self-efficacy and the Common Sense Model informed the phases of identifying and defining common themes across interviews. LT and LJ presented codes and themes to the rest of the author team who contributed to the interpretation of the results. LT, AK (Professor, Ph.D.), and JH (Professor, Ph.D.) are part of the group that developed GLA:D^®^ Back and is training clinicians in the program. SR (Professor, Ph.D.) and LJ have extensive experience in qualitative research but had no prior connection to GLA:D^®^ Back or back pain research. This meant that the interpretation was performed from the perspectives of both in-depth understanding of the intervention and an external view on back pain care.

## Results

### Key themes

The thematic analysis of the data revealed four key themes each characterising a group of patients that perceive and manage LBP differently after having participated in the GLA:D^®^ Back program. In presenting the four themes, we stay close to participants’ wording of their experiences and the way they describe how they manage their pain after attending the program. Key themes are accordingly presented under the headings: “Feeling miscasted”, “Maintaining reservations”, “Struggling with habits” and “Handling it”. In the following, patient-numbers refers to Table 1.

#### Feeling miscasted

For the participants in the “feeling miscasted” group, understandings related to pain seemed to be unchanged after participation in GLA:D^®^ Back. These three participants, who were all dissatisfied with GLA:D^®^ Back, understood chronic pain as a threatening sign of severe tissue damage or injury and reported fear of pain and associated suffering, which, associated with limited pain-control coping strategies, leading to avoidance of pain-provoking movements and activities. The GLA:D^®^ Back program did not change their pain perceptions. They felt misplaced or miscasted in the program and that the intensity of their pain was underassessed.

P14: It might be a good program, but I am a complete miscast. It was not made for people like me. I am not able to do these things; I am in too much pain.

They felt that it was very difficult to cope and find new adaptive strategies to live a physically active life. Comparisons with the ‘unproblematic’ body appeared, which created negative emotional states, including feelings of frustration and hopelessness:

P13: It's not such a life as I had before, before I got low back pain. Before, I was very active, always doing sports.

P15: It’s that fear of doing more damage to my… because if I damage it, I’m not going to be able to walk, I’m not going to be able to play with my child, and what if I’m disabled and can’t do anything?

P13: What if I can’t do my work? It terrifies me. I don’t know if I am going to be able to cope with it in the future, physically. All I can think about is how much I hurt and if the suffering will ever end.

Because of the daily pain, these participants reported that the body could not be trusted to perform daily physical activities. They felt different to other people because of their condition. Avoidance towards certain physical activities was often acknowledged largely due to the fear of increased pain during or after physical activity:

P14: Yes, I certainly avoid some situations because of the fear of increased pain. Yes, definitely! We have a four-storey house, so I really try to plan my day to avoid having to climb the stairs too many times. I try to avoid vacuuming as well because it causes too much pain.

Moreover, participants reported a fear of mistrusting their physical capacity, and a fear of not being able to cope with their own bodily signals during physical activities. By taking an inventory of physical abilities, activities had to be prioritised and planned a long time in advance. Most participants only participated in very special or highly important activities, which could be viewed as an active and reflective choice:

P13: I used to be very active. But now… If I do something one day, I always have to consider: Is the physical activity really worth it?

The participants had knowledge about the health benefits of being physically active, and often contemplated the disadvantages of being physically inactive, in fact they longed to live a life in which they were healthier and more physically active. Despite this knowledge, they pointed to the many obstacles to physical activity, and expressed a strong distrust for future activity plans. The feeling of having a malfunctioning body that they mistrusted overshadowed their cognitive and emotional attention:

P13: I think this is problematic. I have diabetes, so being physical active is important. But I just can’t do it – it causes too much pain. Yes, now the body is problematic. I cannot participate in the things other people do and things that are important to my health condition. It is there all the time in the back of my head.

P15: I would love to do the exercises, but they simply put me in a wrong group. It is really frustrating. But this program is not made for people like me. I am not physical capable of doing these exercises. It is way too painful. It is not wise to do it when it hurts so much.

Participants also pondered over the fear of being a burden to their relatives or others. Being as independent as possible was viewed as an ultimate goal. Because of changing life circumstances, these participants felt that their lives became vulnerable, and even out of control, and that their bodies prevented them from living a normal life. Being physically unable to perform work or having to take sick‐leave or early retirement due to sickness, tended to create feelings of inferiority and disconnection from society.

In summary, participants in this group had not changed their understanding of their illness. Rather, they felt they were cases of LBP that did not fit with the understanding of LBP as presented GLA:D^®^ Back. They had not increased nor achieved a new sense of control in their life after having participated in the intervention program.

#### Maintaining reservations

Two participants, not satisfied with GLA:D^®^ Back, presented pain experiences challenging that movement is a sensible strategy in coping with LBP.They were unable to incorporate messages from the program into their behaviour demonstrating how uncertainty can arise from personal accounts of one’s experiences. These two participants seem to experience a mismatch between the messages they received regarding LBP and the events of pain perception they experience.

P11: There are situations when I’m doing my exercises, then suddenly my back will be hurting. I haven’t done anything unusual, but the pain is there. Then I start to get doubts: what if this is not doing any good after all?

P12: I have done everything they told me to do, but the pain keeps coming back. There is no improvement. I know it won’t kill me, but … somehow I don’t really think the clinicians know what pain is.

They tried to incorporate new knowledge about pain when assessing their situation, but found it difficult to focus on this, as new pain experiences evoked a sceptical attitude towards the understanding of pain presented in the program.

In summary, participants in this group seem to be open to changing their illness understanding relating to LBP. They might, to a certain degree, have developed a new understanding of LBP, but if so, it did not influence their pain perception and it did not inform new strategies for managing pain. Nor do these patients indicate that their self-efficacy had increased.

#### Struggling with habits

Six of the participants, satisfied or somehow satisfied with GLA:D^®^ Back, managed to internalise new understandings of LBP but were not able to change behaviour. Much of their pain-related behaviour is described as habitual and being automatized. The participants know that it would be beneficial to change their behaviour but are confronted with the challenge to change their habits.

P01: I know now that my pain isn’t dangerous, but it has become some kind of habit to avoid situations that might provoke pain. Not because I fear harm but because it has such a natural thing to me to avoid certain movements and activities that I don’t really think about it anymore. It is difficult to change that behaviour.

P06: A sedentary lifestyle is not helping me, but I find it really hard to motivate myself. For some reason the couch always seems more appealing.

P09: I experience back pain, but there is nothing wrong with my body. It is more a mental barrier that I avoid physical activity. It is not harmful, on the contrary, it is beneficial, but the pain that accompanies it is still very unpleasant, so I avoid it.

In summary, these participants understood that LBP should not keep them from being active, and they also express a perceived ability to influence their situation, i.e. apparently high levels of self-efficacy. They know what they should do and feel they can do it. Still, they do not, at this point, succeed in changing their actual management of physical activity in everyday life because habitual behaviours tend to over-rule this understanding.

#### Handling it

For four of the participants, the transformation was immense, and they were very satisfied with GLA:D^®^ Back. They went from experiencing LBP as detrimental for living a good life before attending the program, to perceiving it as more insignificant after.

P02: I have realized that you can’t let the pain dominate your life. You’ve just got to keep going.

P04: If chronic pain doesn’t mean more harm and there aren’t any magical medical answers, what’s left to do? Accept the pain as the new normal, adapt to it, and learn how to manage it.

These participants expressed that they needed physical activity, and, more importantly, they felt they were now capable of carrying out physical activities despite the pain. These participants felt that it was important to demonstrate to themselves that their body was functioning. They mentioned their willingness to take part in physical activities.

P03: I try to walk each day even though my body hurts. While it may seem counterintuitive, movement helps reduce my pain and improves conditioning.

P02: I really enjoy riding my bike now even though it might hurt. By being able to tell the difference between acute pain and chronic pain, I have changed how I react to my back pain by not being so guarded or worried about it.

A hopeful attitude and a spirit towards engaging in activities in daily life were apparent. They focused mainly on the active self and the capable body. Maintenance of physical activities was experienced as an important element for improved health and well‐being:

P05: If I see the slightest chance to make it, then I do it! I have, in the past, avoided certain situations, although I do not think of that anymore! Instead, I now reflect on the good things! I always remind myself: It may hurt, but it won’t kill you.

P04: Participating in the program has helped me live my life, coping with the pain, running a house, looking after my family and working part time.

They placed emphasis on continuously evaluating what worked well. They focused on developing individualized strategies that worked best for them in specific situations, enabling them to be active:

P03: Why make things harder than what they are? Heavy lifting, for example, makes my pain worse. So instead of carrying a heavy load in one trip, I divide it into lighter loads and make multiple trips.

In summary, this group changed how LBP influenced their everyday life. They changed their understanding of LBP as well as achieved a new sense of control in the way they relate to and involve physical activity. Importantly, when they use this newly-gained understanding in their everyday life, their pain perceptions and experiences of events confirm their understanding of LBP. Thus, a positive loop of changed understanding and behaviours may have been initiated.

## Discussion

Four key themes identified in the thematic analysis highlight that patient interpret and use evidence-based messages regarding LBP differently depending on the experiences gained and their individual interpretations of these. Their interpretations influenced the way LBP was understood and managed after participation in a structured program of patient education and exercises aiming at increasing self-management.

During interviews, all participants highlighted that physical activity is important. With different emphasis they highlighted the importance of physical activity for their general health, well-being and/or quality of life. They also highlighted how LBP had prevented them from participating unhampered in everyday physical activities and that this led to feelings of confinement and isolation, and for some participants this was accompanied by a persistent feeling of helplessness. Some participants were anxious if they would be a burden and lose their job. Such concerns were presented although the study was conducted in the context of a Scandinavian welfare system, where people unable to work are financially supported and may indicate that work participation is an important part of personal identity and connection to society. Previous studies have described how people with pain conditions affecting work ability express a perceived threat to their self-esteem and a sense of letting their colleagues down [25, 26].

Before attending the GLA:D^®^ Back program all participants in this study described that they had tended to focus on their fragile physical and emotional state, while also reporting the desire to be more physical active.

For the patient in group c and d (“Struggling with habits” and “Handling it”), the GLA:D^®^ Back program facilitated a transition from a threatening LBP representation and maladaptive body awareness to a nonthreatening representation and an adaptive body awareness. For these two groups the intervention resulted in a reduction in fear experiences. For the participants in group d it seems that participation in GLA:D^®^ Back resulted in new abilities to cope with everyday challenges and being active presented the outcome of a new representation of their illness. A connection which is depicted in the Common Sense Model [11]. However, results also revealed that some of the participants (group a “Feeling miscasted” and b “Maintaining reservations”) did not experience that movement is beneficial or that they became able to engage in new activities. These patients either did not change their understanding of LBP at all (group a) or did not have an emerging change reinforced by personal experience (group b). In between participants presenting immense positive changes and those presenting a lack of changes, we found that some patients (primarily group c “Struggling with habits”) did change their understanding of LBP but they were not successful in changing their behaviour. Thus, they appeared to develop more positive perceptions of LBP and self-efficacy for being capable of managing their condition, but were unable to translate this into behaviour change illustrating that behavioural change depends not only on capability but also on other aspects such as motivation and opportunity [27]. The proportional representation of patients in groups a to d did not reflect the GLA:D^®^ Back patient population as we oversampled patients who were dissatisfied to ensure their perspectives. Two thirds of the study sample was satisfied whereas this is reported by eight out of ten in the full patient population [28].

During interviews, participants repeated the messages clinicians had presented in the program. Although this represented a new understanding for some, the confidence in a positive outcome was not without reluctance for all. The understandings of and confidence to the messages were different. Some of the participants were able to internalise the messages (c and d). Others (a and b), however, repeated them without being able to translate them into a change in behaviour and some even distanced themselves from the messages.

A key strength of this study is the inclusion of participants with both positive and negative experiences and attitudes to the GLA:D^®^ Back program. The purposive sampling was set up so that findings would expectably represent a broad spectrum of patients’ perspectives in preference to focusing on the overall dominant perspectives and/or most valuable outcome. The study focuses on illuminating differences in patients’ reception of an intervention and different kinds and degrees of impact on the participants’ everyday life living with LBP. Despite oversampling, only few study participants represented views of those not satisfied and data saturation was unlikely reached for the aspects related to group a and group b. Other key themes might have emerged with a larger sample size.

Clearly, not all patients were met with what matched their needs and pre-perceptions. It is likely that this type of intervention is not effective for all patients even though they fulfil the medical inclusion criteria, or perhaps delivery of the intervention was not sufficiently adapted to their individual needs. An individualized cognitive approach to supporting patients with LBP to regain trust in physical activity was recently found more effective than a group-based program with no individualization [29]. The GLA:D^®^ Back program has options for individualization within the group-based structure, but it is unknown how this was operationalized in the delivery of the program in individual clinics. Both GLA:D^®^ Back and the individualized cognitive approach aim to help people build self-efficacy potentially by using vicarious and personalized experiences as sources [19, 30]. A group-based program would provide better options for providing vicarious experiences but may suffer from less time to help individual patients reflect on their personal experience and emotional reactions. At this point it is unknown how to potentially identify patients more likely to benefit from one or the other approach.

Our results support previous findings that pain related disability is linked to the way pain is understood by individual patients thereby supporting the relevance of the Common Sense Model [14, 17, 31]. It warrants further investigations to understand how pain cognitions are most effectively affected. This would involve investigating how patients’ readiness to change when seeking care might be appreciated and dealt with [32, 33], the impact of delivery style on different patient groups (e.g., group-based versus individual or clinician delivered versus digital), and the how fidelity in treatment delivery affects outcomes [34] Investigating the delivery of GLA:D^®^ Back and similar interventions would inform if specific clinician training related to e.g. particular behavioral change techniques or communication techniques may be needed to meet the patients who expressed a lack of trust in the information conveyed during the program.

It should also be recognized that patients’ perceptions were studied within a specific setting of Danish private practice and the relevance to other clinical settings or other countries, including low- and middle-income countries cannot be deduced from the study.

## Conclusion

A structured program of patient education integrated with exercises (GLA:D^®^ Back) for people with chronic LBP was experienced very differently by different people. Our results suggest that that the impact of having participated in theprogram relate to how the content of the program resonated with the individual patient's experiences and prior understanding of LBP. Not all patients changed their understanding or came to internalise new understandings during a 10-weeks program, however the results support existing evidence that an improved understanding of what LBP may translate into people being less negatively affected. Awareness of the ways individuals’ understanding of LBP interact with behaviour and physical activities appear central for providing adaptive professional support and meeting the individual needs.

## Supplementary Information

**Additional file 1.** Interview Guide.

## Data Availability

The data is available from the corresponding author on reasonable request.

## Abbreviations

COREQ: Consolidated criteria for reporting qualitative research
GLA:D: Good life with osteoarthritis from Denmark. Is used for the GLA:D^®^ Knee/Hip program not for GLA:D^®^ Back
LBP: Low back pain
