# Supplementary material for: Pain cognitions and impact of low back pain after participation in a self-management program: a qualitative study
Source: Chiropr Man Therap. 2022 Feb 21;30:8. doi: 10.1186/s12998-022-00416-6 (PMC8862196; doi:10.1186/s12998-022-00416-6)
Supplement: Supplementary file 1 — Additional file 1. Interview Guide. [file 12998_2022_416_MOESM1_ESM.docx]

Interview guide translated from Danish.

Intro: Inform participant that the interview is meant to provide knowledge about you who have experience with back trouble and about your thoughts about the GLA:D Back program that you participated in.

| **Theme** | | **Topics** |
| --- | --- | --- |
| ***Profile*** | | Person characteristics: Demographics, education, family |
|  | | Reason for entering GLA:D Back  Ways that back pain affects life |
|  | | Expectations for future related to back pain |
|  | | Openness about having back pain |
|  | |  |
| ***Physical Activity*** | | How, when, with whom  PA changes with back pain |
|  | | What being active means to participant |
|  | | Use of exercises from the program |
|  |  |  |
| ***Pain Knowledge*** | Things affecting if it is a good/bad day in relation to back pain |  |
|  | Causal beliefs, what is wrong  What believed happens when having pain |  |
|  | |  |
| ***Coping*** | | Adjusting everyday life to pain  Behaviours adopted to take care of the back / to reduce pain |
|  | | Perceived pain control  Experienced relation between pain and behaviour |
|  | | Actions / behaviours when experiencing pain. Any change over time?  Changed everyday life? |
|  | | Specific experience with anything worsening or improving back pain |
|  |  |  |
| ***Experience with GLA:D Back*** | Overall experience |  |
|  | Main outcome |  |
|  | | Negative aspects |
|  | | Things lacking in program |
|  | | Experience with group-based |
|  | | Motivated for sessions? Development over time |
|  | | Experience with physical parts  Experience with theoretical parts |
|  | | Knowledge use during exercises |
|  | | The role of the clinician |
|  | | Achieved tools for self-management |
|  | |  |
| **Round-off** | | Anything else |
